# Sex-specific clinical characteristics and prognosis of coronavirus disease-19 infection in Wuhan, China: A retrospective study of 168 severe patients

**DOI:** 10.1371/journal.ppat.1008520

**Published:** 2020-04-28

**Authors:** Yifan Meng, Ping Wu, Wanrong Lu, Kui Liu, Ke Ma, Liang Huang, Jiaojiao Cai, Hong Zhang, Yu Qin, Haiying Sun, Wencheng Ding, Lingli Gui, Peng Wu

**Affiliations:** 1 Cancer Biology Research Center (Key Laboratory of the Ministry of Education), Tongji Hospital, Tongji Medical College, Huazhong University of Science and Technology, Wuhan, China; 2 Department of Respiratory and Critical Care Medicine, Tongji Hospital, Tongji Medical College, Huazhong University of Science and Technology, Wuhan, China; 3 Department of Infectious Diseases, Tongji Hospital, Tongji Medical College, Huazhong University of Science and Technology, Wuhan, China; 4 Department of Hematology, Tongji Hospital, Tongji Medical College, Huazhong University of Science and Technology, Wuhan, China; 5 Department of Gynecologic Oncology, Tongji Hospital, Tongji Medical College, Huazhong University of Science and Technology, Wuhan, China; 6 Department of Anesthesiology, Tongji Hospital, Tongji Medical College, Huazhong University of Science and Technology, Wuhan, China; University of Iowa, UNITED STATES

## Abstract

To confirm the relationship between sex and the progression of Coronavirus Disease-19 (COVID-19), and its potential mechanism, among severe patients. For this retrospective study, we included 168 consecutive severe patients with pathogen-confirmed COVID-19 who were hospitalized between January 16th and February 4th, 2020, at Tongji Hospital in Wuhan, China. Clinical characteristics, laboratory parameters, and outcomes were compared and analyzed between males and females. In the present study, we analyzed 168 severe patients with COVID-19, including 86 males and 82 females, and 48 patients (28.6%) were diagnosed as critically ill. Of 86 male patients, 12.8% (11/86) died and 75.6% (65/86) were discharged; of 82 female patients, 7.3% (6/82) died and 86.6% (71/82) were discharged. Eleven laboratory parameters showed significant differences between male and female patients, and six of them were higher during the whole clinical course in patients who died than in patients who were discharged. In adjusted logistic regression analysis, males with comorbidities presented a higher risk of being critically ill than males without comorbidities (OR = 3.824, 95% CI = 1.279–11.435). However, this association attenuated to null in female patients (OR = 2.992, 95% CI = 0.937–9.558). A similar sex-specific trend was observed in the relation between age and critically ill conditions. We highlighted sex-specific differences in clinical characteristics and prognosis. Male patients appeared to be more susceptible to age and comorbidities. Sex is an important biological variable that should be considered in the prevention and treatment of COVID-19.

## Author summary

Overall, our results highlight sex-specific differences in clinical characteristics and prognosis to COVID-19, which are consistent with the sex-bias observed in other COVID-19 related studies. We also provided more evidence and explored the underlying mechanism with epidemiological analysis. Six candidate laboratory indicators with significant sex differences were considered as potential mediators affecting the prognosis of COVID-19. The age-specific risk distributions of COVID-19 showed a positive correlation between the prognosis and age/ comorbidities, which was significantly affected by sex. We also emphasize that sex is a biological variable that should be considered in the prevention and therapy of COVID-19. It is our hope that these methods may serve as a guide to the clinician in providing timely and specific therapy. Obviously, future studies are warranted to elucidate the different pathways and cellular responses between women and men. The key challenge is how best to use this disparity for providing adequate protection in both males and females.

## Introduction

In December 2019, the first coronavirus disease-19 (COVID-19) cases associated with severe acute respiratory syndrome coronavirus 2 (SARS-CoV-2) were identified in Wuhan, Hubei, China [1–3]. As of March 26^th^, 2020, there have been 465,781 confirmed cases of COVID-19 worldwide including China, Italy, the United States, Spain and Iran, with 50,006 patients were diagnosed in Wuhan, China. More than 20,000 people have died from COVID-19 so far. What also seems to be emerging among these numbers is a clear sex difference in who is contracting and dying from the virus. Several epidemiological studies observed possible differences in the progression of disease between different sexes [4–9]. Compared to women, it is suggested that men are more likely to be admitted to intensive care units and are also associated with higher mortality. Therefore, to confirm and further explore the possible mechanisms of the sex differences in clinical characteristics of COVID-19 patients, we conducted a single-center retrospective analysis in Tongji Hospital, Wuhan, China. In total, 168 severe cases were included. We highlighted sex-specific differences in the clinical characteristics and prognosis of COVID-19, which are consistent with the sex bias observed in other COVID-19-related studies. We also provided more evidence and explored the underlying mechanism with epidemiological analysis.

## Results

**Fig 1:**
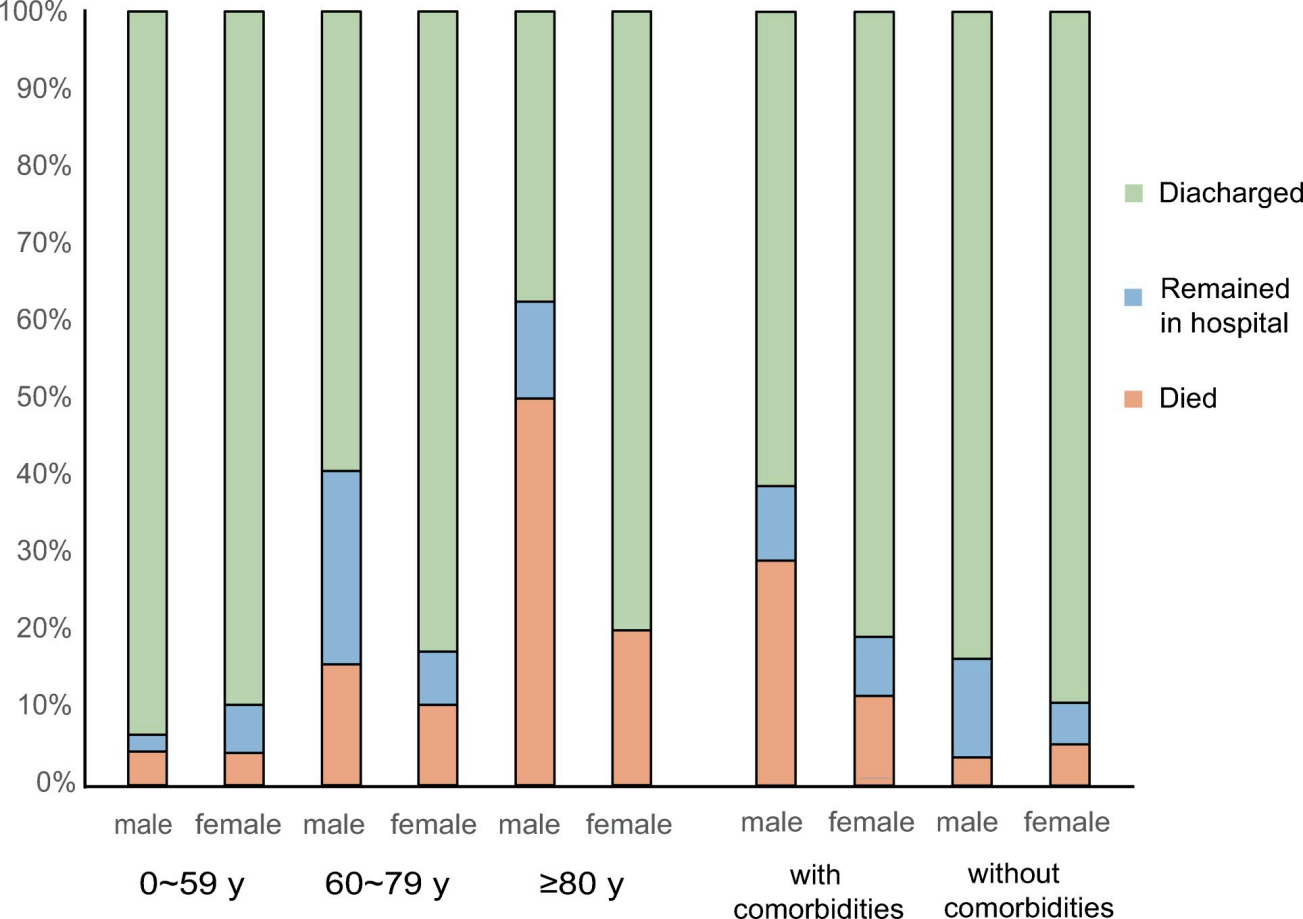
The sex-specific clinical outcomes between different age groups and with/without comorbidities.

In this study, we performed a retrospective analysis on 168 severe or critically ill patients with pathogen-confirmed COVID-19. The study sample comprised of 86 males and 82 females. Outcomes were assessed as discharged, remaining in hospital, or death. Fig 1 illustrates the proportion of males and females with different clinical outcomes between different age groups and in patients with/without comorbidities. In total, 17 of the 168 patients (8.9%) with confirmed COVID-19 died following progression. 136 patients survived to hospital discharge, giving a survival to hospital discharge rate of 81.0%. Significant differences existed across different age groups with regards to mortality rates (*P =* .0001) and hospital discharge rates (*P =* .0014). When comparing the mortality and hospital discharge rates between women and men, men had a trend toward a higher risk of mortality and a lower hospital discharge rate (Fig 1). Furthermore, what stands out in this chart is that the patients with underlying diseases had a higher mortality rate than those who did not (*P <* .0001), which implies that underlying diseases might influence the severity of COVID-19 (Fig 1).To figure out how sex interacts with these comorbidities, we have graphed and analyzed the outcome data of male and female patients with and without comorbidities separately. Fig 1 indicated that males with comorbidity had the highest mortality rate among all patients.

Table 1 shows the sex-specific baseline characteristics of 168 severe patients infected with COVID-19. Of the 168 patients, the median duration from illness onset to pathogens identified and hospital admission was 9 days (IQR 7–11.5) and 7 days (IQR 7.0–11.0), respectively. Fifty-seven (33.7%) patients had comorbid diseases (Table 1). On admission, 156 patients (92.9%) had fever as the initial symptom, and 121 patients (72.0%) had cough. Other main symptoms included expectoration (41.7%), fatigue (38.7%), dyspnea (35.1%), myalgia (28.6%), diarrhea (26.2%), headache (13.1%), and nausea (10.7%, Table 1). Among all signs and symptoms, only headache showed significant differences between males and females (*P =* .016).

**Table 1:** The sex-specific baseline characteristics of 168 severe patients infected with COVID-19.

	All patients (n = 168)	Male (n = 86)	Female (n = 82)	*P* value^a^
**Age (y, mean, [SD])**	56.7(15.1)	56.9(15.8)	56.4(14.4)	.82
Age groups				
0 to 59	94(56.0%)	46(53.5%)	48(58.5%)	.68
60 to 79	61(36.3%)	32(37.2%)	29(35.4%)	. . .
≥80	13(7.7%)	8(9.3%)	5(6.1%)	. . .
Medical staff	10(6.0%)	4(4.7%)	6(7.3%)	.53
Systolic pressure (mmHg, mean, [SD])	126.7(17.5)	129.4(18.1)	123.7(16.4)	.048*
Diastolic pressure (mmHg, mean, [SD])	80.3(11.9)	81.7(11.7)	78.8(12.0)	.12
Pulse (times/min, mean, [SD])	90.9(16.5)	92.5(17.9)	89.1(14.8)	.21
**Onset of symptom to (d, median, [IQR])**				
Hospital admission	9(7–11)	10(7–11)	8(5–10)	.069
Death	21(15–24)	16(15–22)	24(23–27)	.023*
Discharge	34(30–42)	33(30–40)	34(28–43)	.96
Pathogens identified	9(7–11.5)	9(7–11)	9(6–12)	.77
**Comorbidities**	57(33.9%)	31(36.1%)	26(31.7%)	.55
Hypertension	41(24.4%)	20(23.3%)	21(25.6%)	.72
Diabetes	20(11.9%)	11(12.8%)	9(11.0%)	.72
Cardiovascular disease	9(5.4%)	6(7.0%)	3(3.7%)	.50
Chronic kidney disease	2(1.2%)	2(2.3%)	0(0%)	.50
Cerebrovascular disease	2(1.2%)	2(2.3%)	0(0%)	.50
COPD	1(0.6%)	1(1.2%)	0(0%)	1.00
Malignancy	1(0.6%)	1(1.2%)	0(0%)	1.00
**Signs and symptoms**				
Fever	156(92.9%)	82(95.3%)	74(90.2%)	.20
Cough	121(72.0%)	66(76.7%)	55(67.1%)	.16
Expectoration	70(41.7%)	38(44.2%)	32(39.0%)	.50
Fatigue	65(38.7%)	33(38.3%)	32(39.0%)	.93
Dyspnea	59(35.1%)	32(37.2%)	27(32.9%)	.56
Myalgia	48(28.6%)	25(29.1%)	23(28.1%)	.88
Diarrhea	44(26.2%)	20(23.3%)	24(29.2%)	.38
Headache	22(13.1%)	6(7.0%)	16(19.5%)	.016*
Nausea	18(10.7%)	7(8.1%)	11(13.4%)	.27
Anorexia	15(8.9%)	6(7.0%)	9(11.0%)	.36
Vomiting	15(8.9%)	7(8.1%)	8(9.8%)	.71
Thoracodynia	12(7.1%)	6(7.0%)	6(7.3%)	.93
Abdominal pain	7(4.2%)	4(4.7%)	3(3.7%)	1.00
Pharyngalgia	7(4.2%)	5(5.8%)	2(2.4%)	.44
Dizziness	7(4.2%)	2(2.3%)	5(6.1%)	.27
Haemoptysis	3(1.8%)	3(3.5%)	0(0.0%)	.25
Pharyngeal hyperaemia	3(1.8%)	2(2.3%)	1(1.2%)	1.00

Abbreviations: COPD, chronic obstructive pulmonary disease; IQR, interquartile range; SD, standard deviation.

^a^*P* values comparing male and female are from *χ*^*2*^ test, Fisher’s exact test, *t* test or Mann-Whitney U test.

*Significant at *P* < .05.

**Fig 2:**
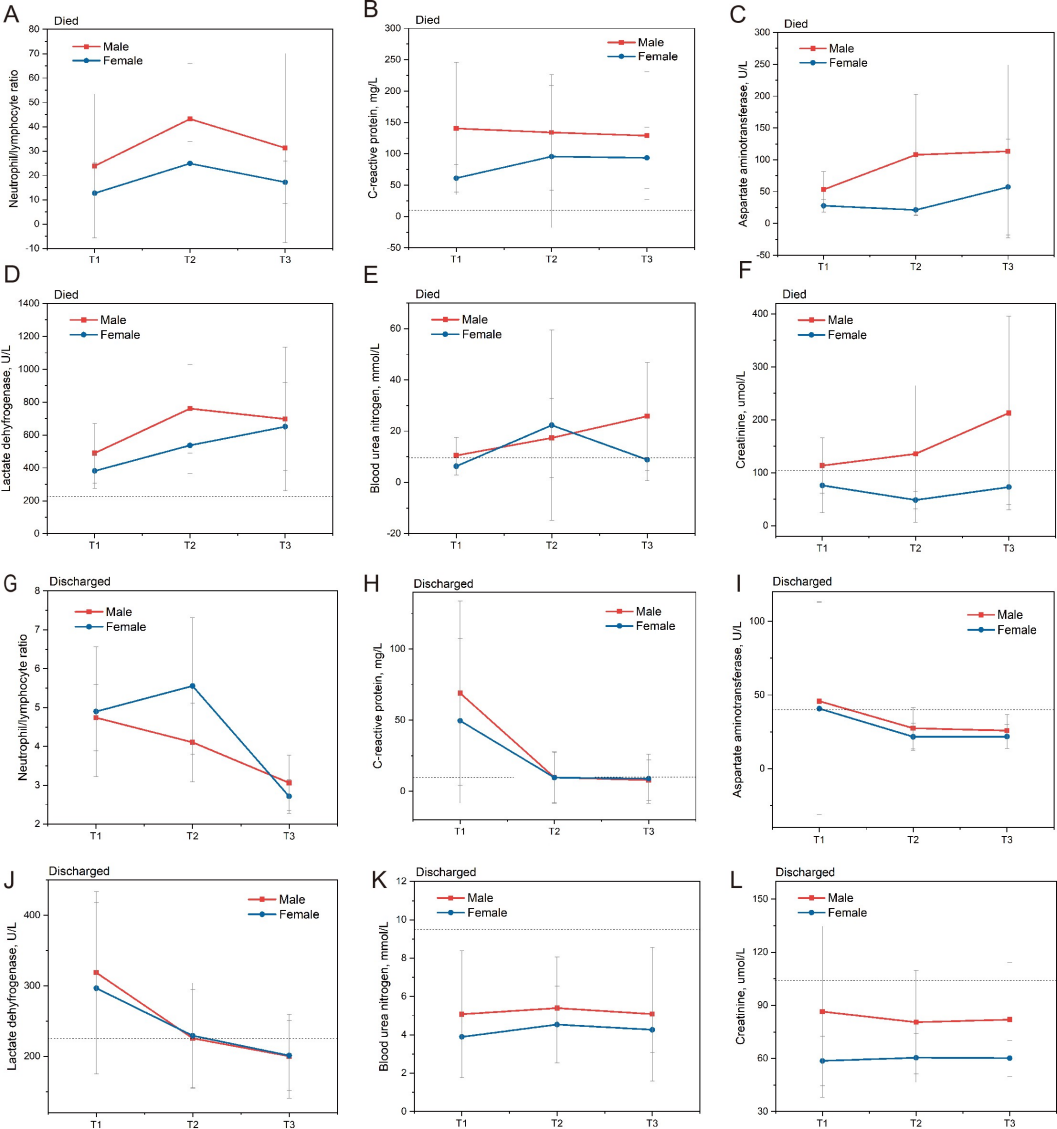
The sex-specific dynamic changes in selected laboratory parameters during the course of COVID-19. (A), the sex-specific dynamic change in the neutrophil-to-lymphocyte ratio in patients who died; (B), the sex-specific dynamic change in C-reactive protein in patients who died; (C), the sex-specific dynamic change in aspartate aminotransferase in patients who died; (D), the sex-specific dynamic change in lactate dehydrogenase in patients who died; (E), the sex-specific dynamic change in blood urea nitrogen in patients who died; (F), the sex-specific dynamic change in creatinine in patients who died. (G), the sex-specific dynamic change in the neutrophil-to-lymphocyte ratio in patients who were discharged; (H), the sex-specific dynamic change in C-reactive protein in patients who were discharged; (I), the sex-specific dynamic change in aspartate aminotransferase in patients who were discharged; (J), the sex-specific dynamic change in lactate dehydrogenase in patients who were discharged; (K), the sex-specific dynamic change in blood urea nitrogen in patients who were discharged; (L), the sex-specific dynamic change in creatinine in patients who were discharged. T1, the first test after hospital admission; T2, the midpoint test during the whole hospital stay; T3, the last test before discharge. The dashed lines in gray show the upper limit of normal of each parameter.

On admission, the blood routine workup of most severe COVID-19 patients showed lymphopenia (lymphocyte count <1.1 × 10⁹/L; 112/168, 66.7%), which was consistent with the finding of previous studies [4–9]. Eleven laboratory parameters showed significant differences between male and female patients, especially for inflammatory markers and functional indexes of the liver and kidney. All of them were increased substantially in male patients (*P <* .05, Table 2). Six clinical laboratory parameters, including hematological and biochemical parameters, were chosen from indicators with significant differences between patients who died and in patients who were discharged. To determine the potential effects of indicators with sex differences, we tracked the sex-specific dynamic changes of the six selected laboratory parameters during the course of COVID-19 (Fig 2). Clinical data from 153 patients with complete clinical courses were analyzed, including 17 patients who died and 136 patients discharged from the hospital. T1 refers to the first test after hospital admission, T2 refers to the midpoint test during the whole hospital stay, and T3 refers to the last test before discharge. During hospitalization, patients who died had a marked increase in the neutrophil-to-lymphocyte ratio (NLR) at admission and even developed a higher NLR over time. Similarly, C-reactive protein (CRP), aspartate aminotransferase (AST), lactate dehydrogenase (LDH), blood urea nitrogen (BUN), and serum creatinine were all higher in patients who died than in patients discharged from hospital (*P <* .05). Therefore, we analyzed the sex-specific dynamic changes in selected laboratory parameters during the course of COVID-19 in patients who died (Fig 2A–2F) and in patients who were discharged (Fig 2G–2L). As shown in Fig 2A–2F, there were clear separations between the curve for males and the curve for females in patients who died except for BUN. As the clinical status deteriorated, the levels of these parameters progressively increased before death. Furthermore, the sex-specific curves for NLR, CRP, AST and LDH crossed each other in discharged patients, whereas BUN and creatinine were separate (Fig 2G–2J).

**Table 2:** The sex-specific laboratory parameters of 168 severe patients infected with COVID-19.

Laboratory parameters	All patients (n = 168)	Male (n = 86)	Female (n = 82)	*P* value^a^
White blood cell count (×10⁹/L, normal range 4–10,median, [IQR])	5.2(3.9–6.9)	5.3(4.0–7.5)	5.0(3.9–6.4)	.30
<4	50(29.8%)	23(26.7%)	27(32.9%)	.68
4–10	109(64.9%)	58(67.4%)	51(62.2%)	. . .
>10	9(5.4%)	5(5.8%)	4(4.9%)	. . .
Neutrophil count (×10⁹/L, normal range 1.8–6.3, median, [IQR])	3.6(2.5–5.4)	4.0(2.6–5.6)	3.3(2.5–5.0)	.23
Lymphocyte count (×10⁹/L, normal range 1.1–3.2, median, [IQR])	0.9(0.7–1.3)	0.8(0.7–1.2)	1.0(0.7–1.3)	.05
<1.1	112(66.7%)	62(72.1%)	50(61.0%)	.13
≥1.1	56(33.3%)	24(27.9%)	32(39.0%)	. . .
Neutrophil/lymphocyte ratio (median, [IQR])	3.8(2.4–7.2)	4.2(3.0–8.2)	3.1(2.2–5.7)	.048*
Monocyte count (×10⁹/L, normal range 0.1–0.6, median, [IQR])	0.4(0.3–0.5)	0.4(0.3–0.5)	0.4(0.3–0.5)	.44
Haemoglobin (g/L, male normal range 130–175, female normal range 115–150, median, [IQR])	130.0(119.0–139)	137.0(131.0–144.0)	120.0(113.0–128.0)	< .0001*
Hematocrit (%, male normal range 40–50, female normal range 35–45, median, [IQR])	38.2(34.5–40.5)	39.8(37.9–41.4)	35.7(32.7–38.6)	< .0001*
Platelet count (×10⁹/L, normal range 125–350, median, [IQR])	188.0(144.0–239.0)	184.0(142.0–239.0)	198.5(150.0–244.0)	.39
<125	27(16.1%)	17(19.8%)	10(12.2%)	.18
Glycosylated hemoglobin (%, normal range 4–6, median, [IQR])	6.0(5.6–6.5)	5.9(5.5–6.7)	6.1(5.7–6.5)	.62
Prothrombin time (s, normal range 11.5–14.5, median, [IQR])	14.1(13.4–14.8)	14.3(13.4–15.0)	14.0(13.4–14.5)	.10
Activated partial thromboplastin time (s, normal range 29–42, median, [IQR])	41.9(37.1–45.6)	43.6(39.1–46.5)	39.6(36.0–43.6)	.013
D-dimer (ug/ml, normal range <0.5, median, [IQR])	0.7(0.4–1.9)	0.8(0.4–2.1)	0.7(0.4–1.3)	.69
≥0.5	108(64.3%)	55(64.0%)	53(64.6%)	.93
Ferritin (ng/mL, normal range 15–150, median, [IQR])	672.4(380.4–1196.6)	884.9(590.0–1478.7)	469.1(286.0–771.3)	< .0001*
Alanine aminotransferase (U/L, normal range ≤40, median median, [IQR])	24.0(15.0–40.0)	31.5(19.0–48.0)	18.0(12.0–31.0)	< .0001*
>40	41(24.4%)	29(33.7%)	12(14.6%)	.0040*
Aspartate aminotransferase (U/L, normal range ≤40, median, [IQR])	30.0(21.5–46.5)	37.5(25.0–52.0)	25.0(19.0–33.0)	< .0001*
>40	54(32.1%)	39(45.4%)	15(18.3%)	.0002*
Albumin (g/L, normal range 35–52, median, [IQR])	34.7(31.2–38.8)	34.3(31.1–38.0)	35.4(31.2–39.0)	.87
Total bilirubin (μmol/L, normal range 3.4–17.1, median, [IQR])	8.5(5.6–11.4)	9.7(7.0–12.7)	7.4(5.0–9.6)	< .0001*
>17.1	18(10.7%)	14(16.3%)	4(4.9%)	.017
Lactate dehydrogenase (U/L, normal range 135–225, median, [IQR])	292.0(225.0–397.0)	305.0(238.0–455.0)	278.5(213.0–351.0)	.050
>225	126(75.0%)	69(80.2%)	57(69.5%)	.11
Blood urea nitrogen (mmol/L, male normal range 3.6–9.5, female normal range 1.7–8.3, median, [IQR])	4.4(3.4–5.7)	4.8(4.0–6.5)	3.9(3.0–4.9)	< .0001*
Creatinine (U/L, normal range 44–133, median, [IQR])	69.5(57.0–81.5)	78.5(71.0–90.0)	58.0(52.0–66.0)	< .0001*
>133	7(4.1%)	6(7.0%)	1(1.2%)	.12
C-reactive protein (mg/L, ≥10 suggests inflammation or infection, median, [IQR])	42.3(12.2–100.3)	59.5(24.8–138.5)	28.7(7.1–73.1)	< .0001*
≥10	136(81.0%)	78(90.7%)	58(70.7%)	.0010*
Glucose (mmol/L, normal range 4.11–6.1, median, [IQR])	6.7(5.7–8.6)	7.1(6.1–8.6)	6.5(5.5–8.6)	.09
>6.1	108(64.3%)	61(70.9%)	47(57.3%)	.066
Erythrocyte sedimentation rate (mm/h, male normal range 0–15, female normal range 0–20, median, [IQR])	28.0(17.0–55.0)	28.0(17.0–48.0)	28.0(19.0–60.0)	.42
Hypersensitive troponin I (pg/mL, normal range ≤28, median, [IQR])	4.9(2.6–15.1)	4.9(2.8–20.1)	4.7(2.5–12.7)	.31
≤28	152(90.5%)	75(87.2%)	77(93.9%)	.14
>28	16(9.5%)	11(12.8%)	5(6.1%)	. . .
Procalcitonin (ng/mL, ≥0.5 suggests inflammation or infection, median, [IQR])	0.06(0.03–0.15)	0.10(0.05–0.24)	0.04(0.02–0.08)	< .0001*
<0.1	119(64.9%)	44(51.2%)	65(79.3%)	.0018*
≥0.1 to <0.25	37(22.0%)	25(29.1%)	12(14.7%)	. . .
≥0.25 to <0. 5	8(4.8%)	6(7.0%)	2(2.4%)	. . .
≥0.5	14(8.3%)	11(12.8%)	3(3.7%)	. . .

Abbreviations: IQR, interquartile range.

^a^*P* values comparing male and female are from *χ*^*2*^ test, Fisher’s exact test or Mann-Whitney U test.

*Significant at *P* < .05.

**Fig 3:**
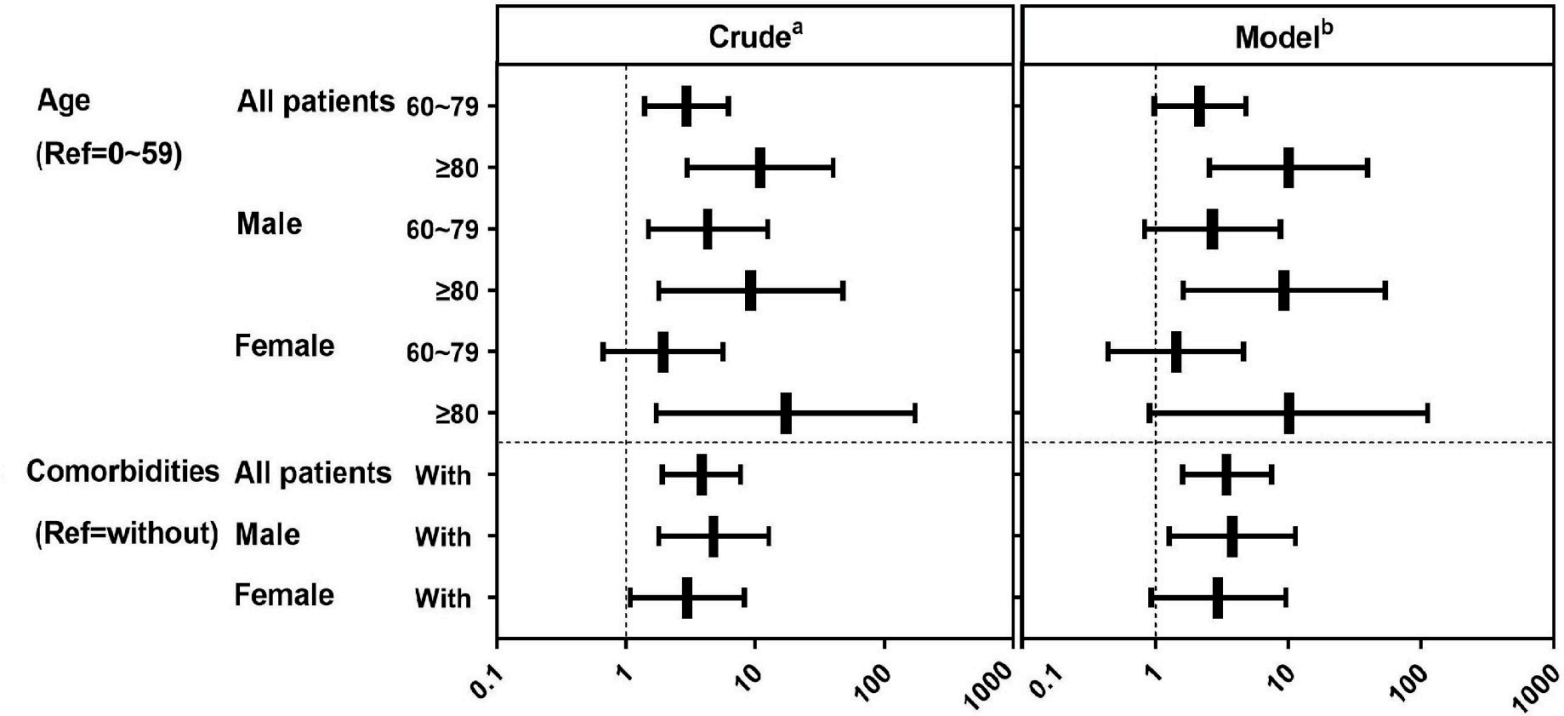
ORs (95% CI) of the rates of critically ill cases according to age and comorbidities. Abbreviations: OR, odds ratio; 95%CI, 95% confidence interval. ^a^Crude: unadjusted. ^b^Model: adjusted for with/without comorbidity(for age) or age(for with/without comorbidity), with/without signs and symptoms of respiratory system, the days from onset of symptom to hospital admission, and the days from onset of symptom to pathogens identified.

In the present study, 48 patients (28.6%) were graded as critically ill cases and were regarded as patients with poor prognosis. The age-specific risk distributions of COVID-19 showed a positive correlation between poor prognosis and age (*P* < .0001). The results from binary logistic regression analysis presented a higher risk of critically ill progression in the elderly aged 80 and above than in young and middle-aged individuals aged < 59 years old (OR = 10.968, 95% CI: 3.005–40.037, Fig 3). In the present analysis, we included comorbidities, respiratory symptoms, days from illness onset to the first admission and pathogens identified as potential confounding factors. After adjustment for confounders, elderly males aged >80 years old were more likely to develop critically ill conditions than males aged <59 years old (OR = 9.333, 95% CI: 1.618–53.845). However, for females, the difference was not significant according to the adjusted model (OR = 10.161, 95% CI: 0.911–113.346). Our analysis also illustrated that the patients with comorbidity had a higher risk of critically ill conditions and mortality rate (*P* < .0001). The relation between comorbidities and critically ill conditions was assessed with appropriate adjustments for covariates including age, respiratory symptoms, days from illness onset to the first admission and the pathogens identified. A similar sex-specific trend was observed (males: OR = 3.824, 95% CI: 1.279–11.435; females: OR = 2.992, 95% CI: 0.937–9.558; Fig 3).

## Discussion

To our knowledge, this report is the first sex-specific analysis of a case series of patients hospitalized with severe COVID-19 to date. Some evidence from single-center studies shows a difference between the incidence rate and fatality rate of COVID-19 among males and females. These studies have identified that men may be more prone to coronavirus infection than women and have a higher risk of poor outcome [4–9]. The same trend was also found for MERS-CoV and SARS-xCoV [10,11]. In this study, we conducted a single-center retrospective analysis of 168 critically ill patients, explicitly evaluating the sex differences in clinical characteristics of COVID-19 patients and confirming the positive correlation between fatality and age. Compared with other studies, the duration of the median follow-up in our study was 27 days (IQR 22–34 days), with accurate follow-up and few missing data. In addition, our study included only severe patients and critically ill patients, who have a poorer prognosis and deserve further attention.

While the epidemiological data of COVID-19 have shown sex bias in the fatality rate and clinical characteristics, the mechanism underlying this difference is still not clear. A variety of factors may cause disparities in sex-specific disease outcomes. For instance, sex-specific disease outcomes may be explained by steroids and the activity of X-linked genes, both of which modulate the immune response to viral infection [12,13]. It is well known that males and females differ in both innate and adaptive immune responses. Sex-specific inflammatory responses caused by the unique mode of inheritance of the X chromosome may also lead to the differences [13]. The latest studies have demonstrated that there is the high expression of angiotensin converting enzyme 2 (ACE2), the putative receptor of SARS-nCoV-2, in the normal lung tissue of Asian men [14]. It has been suggested that 17β-estradiol could downregulate lung ACE2 mRNA and protect females from influenza A virus pathogenesis [15,16]. Previous studies also found higher percentages of SARS-CoV infection in male mice than in female mice and provided mechanistic insights related to estrogen [17].

In this study, we explored the potential mediators by sex-specific epidemiological analysis. Significant differences in certain laboratory parameters were observed between males and females (Table 2). After the exclusion of the recognized sex-specific parameters (e.g., hemoglobin and hematocrit), the remaining indicators were identified as potential biomarker candidates that played a potential role in the sex bias of mortality. During hospitalization, NLR, CRP, AST, LDH, BUN and serum creatinine were all higher in patients who died than in patients discharged from the hospital (*P* < .05). We also analyzed the sex-specific dynamic changes in selected laboratory parameters during the course of COVID-19 in patients who died and in patients who were discharged (Fig 2). It has been indicated that these sex differences in NLR, CRP, AST, LDH, and serum creatinine in patients who died may explain the sex-specific clinical characteristics and outcomes. Based on previous studies, other literature on COVID-19 has emphasized the importance of lymphocyte responses and proinflammatory cytokine storms [18–20]. Additionally, abnormal liver and kidney function have also been observed in other studies [5,21], but it was unclear whether they were due to viral characteristics or drug-induced liver/kidney injury [21,22]. Considering the significant differences in the functional indexes of the liver and kidney, more attention should be paid to the monitoring and evaluation of liver and kidney function during the treatment of critically ill patients.

Moreover, recent studies have reported a consistent association between age and disease severity [4–9]. In the present study, the risk of critically ill conditions was significantly associated with age and comorbidities in the crude model. The positive correlation remained robust in males after adjustment, but the effect was attenuated to null in female patients. In total, the results of this study indicate that although age and comorbidities are both important factors that have substantial influence on the progression of COVID-19, the impact showed pronounced sex-specific differences. We speculated that the age effects mainly reflect the physiological and social changes of aging patients, especially for elderly males. Therefore, disparities between males and females suggest that more attention to elderly males with COVID-19 is needed in both research and treatment. It was suggested that the worse prognosis of COVID-19 was associated with chronic comorbidities as a result of weaker immune functions [23,24]. Considering that 36.0% of male patients had chronic underlying diseases (mainly diabetes and cardiovascular and cerebrovascular diseases), more attention needs to be paid to male patients in poor physical conditions.

Several limitations should be noted in the present study. First, the present study was a retrospective analysis that was performed in a single institution, including only patients who were hospitalized and excluding asymptomatic patients. Second, we included only patients with confirmed COVID-19 cases and excluded patients with suspected but undiagnosed cases in the analyses. However, negative test results should not preclude the possibility of infection[25]. Beyond the association of sex and disease severity, further investigation is needed to understand the clinical characteristics and to perform risk assessments in pregnant women with COVID-19 infection [26,27].

Overall, our results highlight sex-specific differences in the clinical characteristics and prognosis of COVID-19 and are consistent with the sex bias observed in other COVID-19-related studies. We emphasized that sex is a biological variable that should be considered in the prevention and treatment of COVID-19. It is our hope that these methods may serve as a guide to the clinician in providing timely and specific therapy. Obviously, future studies are warranted to elucidate the different pathways and cellular responses between women and men. The key challenge is how best to use this disparity for providing adequate protection in both males and females.

## Material and methods

### Ethics statement

This study was approved by the Ethical Committee of Tongji Hospital of Tongji Medical College at Huazhong University of Science and Technology (TJ-IRB20200311). Written informed consent was not obtained because the data were analyzed retrospectively and anonymously.

### Study design and participants

For this retrospective study, we included 168 consecutive severe patients with COVID-19 who were hospitalized between January 16^th^ and February 4^th^, 2020, at Tongji Hospital in Wuhan, China. The clinical characteristics (such as vital signs, symptoms, and laboratory parameters) were monitored up to March 24^th^, 2020, the final date of follow-up. The median duration of follow-up for all patients was 27 days, with accurate follow-up and few missing data. As one of the designated hospitals, Tongji Hospital is responsible for the treatment for critically ill COVID-19 patients in line with the arrangements of the local government. Therefore, according to the COVID-19 diagnosis and treatment plan issued by the National Health Commission, all patients included were graded as severe cases or critically ill cases. Clinical and demographic data of the confirmed cases were collected from their medical records.

### Procedures

All clinical information (i.e., epidemiological characteristics, medical history, underlying comorbidities, symptoms, signs, laboratory parameters, radiological characteristics, and treatment and outcome data) was obtained by reviewing the medical records of patients. After proper training, the data collection was conducted by two individuals independently. EpiData 3.1 was used for data entry and management. The data entry quality was checked by the double data entry verification method to reduce data entry errors. All patients were identified and classified according to the sixth edition of the COVID-19 diagnosis and treatment plan issued by the National Health Commission. Based on the published guide, doctors in Hubei Province should use CT scans to make a clinical diagnosis of suspected coronavirus infections. Real-time reverse transcriptase polymerase chain reaction (real-time RT-PCR) testing kits were then used to confirm coronavirus infection. In this study, all patients included were verified as positive for SARS-CoV-2 infection in throat swabs or bronchoalveolar lavage fluid analyzed by real-time RT-PCR. The specific operation methods were followed according to the instructions and were consistent with other literature [4,5].

### Definitions

According to the COVID-19 diagnosis and treatment plan mentioned above, the clinical classifications of COVID-19 were classified into four main types: mild cases, moderate cases, severe cases, and critically ill cases. Severe cases are defined as patients who met any of the following criteria: (a) with respiratory distress syndrome, respiratory rates ≥ 30/min; (b) the finger oxygen saturation measured after 5 minutes of rest was ≤93%; and (c) PaO_2_ (the arterial oxygen partial pressure)/FiO_2_ (the inspired oxygen fraction) ≤ 300 mmHg. Critically ill cases are defined as patients who met any of the following criteria: (a) developed respiratory failure requiring intubation, (b) presented with shock, and (c) developed other organ failure or were admitted to the ICU.

### Statistical analysis

All analyses were conducted with SAS software version 9.4. The measurement data were expressed as the mean and standard deviation (SD) or median and interquartile range (IQR) values. The comparison between the two groups was performed by independent group t-tests when the measurement data were normally distributed. Otherwise, Mann-Whitney U tests were applied. Enumeration data were summarized as frequency rates and percentages. Intergroup comparison of enumeration data was performed using chi-squared tests or Fisher’s exact tests. Binary logistic regression models were adopted to analyze the influence of age in different age groups or with/without comorbidity on the risk of progression to critically ill cases. *P* < .05 was considered statistically significant.
